# In vitro culture media type impacts gene expression in the freshwater mussel *Lampsilis siliquoidea* (Bivalvia: Unionidae)

**DOI:** 10.1186/s41065-025-00589-z

**Published:** 2025-11-07

**Authors:** Kaitlin E. Ulin, Alexandra R. Phelps, Chase J. Ellis, Marymegan Daly, Ieva Roznere

**Affiliations:** Department of Evolution, Ecology, and Organismal Biology, The Ohio State University, Columbus, OH USA

**Keywords:** Freshwater mussels, Gene expression, Glochidia, In vitro propagation, Transcriptomics, Unionidae

## Abstract

Artificial propagation is an important conservation technique to mitigate the loss of native freshwater mussel diversity. In vitro propagation is an alternative method of metamorphosing freshwater mussel larvae (glochidia) to juveniles without a host fish, but the methodology is still evolving in its rates of metamorphosis. This study provides the first comparison of gene expression in freshwater mussel glochidia metamorphosed in different culture media: M199, L-15, and M199 supplemented with lipids. Compared to the commonly used M199, 1.2% of the transcriptome of glochidia reared in the other culture media showed significantly altered expression levels. Of these differentially expressed transcripts, 86% were the same regardless of whether glochidia were metamorphosed in the different basal culture medium L-15 or in M199 supplemented with lipids. We discuss the Gene Ontology categories with the highest number of differentially expressed transcripts, as well as those categories that were over-represented in the different culture media compared to the whole transcriptome. Our results suggest that the external environment can have a significant impact on the physiology of metamorphosing glochidia and may potentially impact the health and survival of juveniles.

**Supplementary Information:**

The online version contains supplementary material available at 10.1186/s41065-025-00589-z.

## Introduction

Freshwater mussels in the order Unionida are one of the most endangered taxa in North America, with 43% of the 298 species listed as extinct, critically endangered, endangered, or vulnerable by the International Union for Conservation of Nature [1]. Population declines are attributed to myriad factors, such as habitat destruction and alteration, especially dam construction [2], droughts [3], invasive zebra mussels [4], and, potentially, disease [5]. Furthermore, global stressors, such as climate change and water pollution compound with these local factors to exacerbate population declines [6]. Consequently, freshwater mussels have one of the highest extinction rates of any group of organisms [7].

It is critical to mitigate and reverse population declines because mussels contribute significant aquatic ecosystem services. Mussels are impressive filter feeders that remove large quantities of particles, including chemical pollutants, from the water column [8]. Due to their large filtering capacity, mussels also play an important role in nutrient cycling and storage and their activities can increase amounts of benthic algae, which provide food to other invertebrates [8, 9]). Additionally, mussel shells provide habitat for other organisms [10] and dense mussel beds in a river support more abundant invertebrate communities than similar areas with fewer mussels [11]. Because population declines threaten the ecosystem functions provided by freshwater mussels, there has been increasing interest in captive propagation as a conservation technique to mitigate population loss and establish new populations.

Freshwater mussels have a unique life cycle among bivalves. They have an obligate parasitic larval stage that requires external attachment to a host fish (or salamander, in some cases) to complete metamorphosis to a juvenile [12]. After fertilization, female mussels brood the larvae, called glochidia, in specialized gill tissues. Freshwater mussels have evolved fascinating strategies that attract host fish and increase the chances of larval attachment. For example, some species release small packages of larvae that seem to mimic insect larvae [12, 13] or fish fry [14]. Other species use a mantle lure that resembles prey of the host fish [12]. Once a glochidium attaches to the gills, fins, or scales of the fish, a capsule forms around it, initiating transformation to a juvenile mussel. After metamorphosis is complete, the juvenile falls off the host fish and burrows into the substrate. Although some mussel species can transform on a wide variety of fish species, others are host specialists and only use one or a few host species [15]. The unique life cycle of freshwater mussels also means that their reproductive success relies on the presence of appropriate host fish species in their habitat [16].

Artificial propagation is a key conservation strategy used to mitigate population loss of these endangered animals [17]. Freshwater mussels are successfully propagated in fish hatcheries or specialized mussel propagation facilities [18, 19]. Depending on resources, these facilities use either in vivo or in vitro propagation methods [19, 20]. The more conventional in vivo propagation methods attempt to replicate the natural life cycle in the laboratory by infesting host fish with glochidia extracted from mussels. However, increased interest has focused on in vitro propagation methods, which use liquid culture media that mimic the nutrients and environmental conditions of the host fish, thereby completely bypassing the host fish stage [20, 21]. One of the main advantages of in vitro propagation is the potential to produce larger quantities of juveniles ([22], personal observation). Eliminating host fish also reduces labor associated with fish husbandry. Furthermore, the host fish species may be unknown, or the fish species may be state or federally listed, difficult to obtain, or not suited well for captivity [23]. In vivo methods also pose the risk of over-infestation, which may cause stress or mortality to the fish [23]. Furthermore, in vitro propagation allows for easy microscopic examination of glochidia development [24], enabling investigation of influence of variables such as temperature, pH, and nutrients on metamorphosis rates [25, 26]. Since in vitro propagation appears to produce juveniles that are just as healthy and fit as those produced using in vivo methods [22], there has been increasing interest in improving and expanding in vitro culture methods.

Improving in vitro propagation success requires increasing our knowledge of the larval development of mussels, the effects of nutrients on successful development, and the effects of various culture media. Research on freshwater mussel in vitro propagation has focused on improving and simplifying culture media formulations and refining methods for successful metamorphosis [25, 27–31]. Culture media used for mussel propagation include three main components: a basal liquid medium, host fish or alternative serum, and antibiotics/antimycotics. The most commonly used basal liquid medium is Medium 199 (M199), which contains amino acids, lipids, vitamins, and inorganic salts, and requires CO_2_ supplementation to maintain a constant pH that supports glochidia metamorphosis [20]. M199 is known for its reliability and widespread use in freshwater mussel in vitro propagation, where it has been successfully used for a broad range of mussel species [20].

Although M199 is the most widely-used culture medium for freshwater mussels, it does not support metamorphosis for all species, most notably those with atypically shaped glochidia [32]. Most glochidia are broadly categorized as being either hooked or hookless, do not grow much during metamorphosis, and have valves that close completely when exposed to culture media [33]. The less common axe-head shaped glochidia differ from the hooked and hookless types in that they grow significantly during metamorphosis, and have valves that cannot close completely due to their concave shape [32]. Possibly due to one or both attributes, the axe-head shaped glochidia have not been successfully metamorphosed using M199. Wen et al. [32] were the first to successfully transform the axe-head shaped glochidia of *Potamilus alatus* using Leibovitz’s L-15 Medium (L-15). The L-15 specific compound formulations and concentrations differ between M199 and L-15, and L15 does not require CO_2_ supplementation. Subsequent studies have also successfully used L-15 to transform other freshwater mussel species [29, 30], raising the possibility that L-15 may be a good alternative to M199 in transforming a broad range of freshwater mussel species.

Lipid availability during metamorphosis may be an important factor in metamorphosis success and juvenile survival and growth [34, 35]. Previous studies have shown that lipid content in adults is associated seasonally with their reproductive cycle, potentially due to the investment of lipids in brooding glochidia [36]. Juvenile lipid reserves may vary depending on the host fish that they utilize for metamorphosis [37]. Since commercially available culture media, such as M199 and L-15, contain no lipids, these need to be added as a supplementation. Exploration of the effects of lipid supplementation to culture media is needed to improve the success of freshwater mussel in vitro propagation and to increase our understanding of the relationship between developmental transitions and lipid availability for freshwater mussels.

Genomic tools provide a novel approach to improving in vitro propagation methods by providing insight into the cellular processes of an organism during development [38, 39]. The transcriptome, a snapshot of all the genes expressed in an organism at a specific point in time, can show precursors to proteins that respond to changes in environmental and physiological conditions [40, 41]. Because transcriptomics can reveal changes in gene expression associated with metabolism [42] and growth [43], it may be helpful in studying the effects of different culture media types on glochidia development in vitro. Exploring differences in gene expression between glochidia metamorphosed in different culture media types could provide a better understanding of the effects of various media formulations and provide foundational knowledge needed to improve the conservation of highly imperiled and ecologically important freshwater mussels. Although transcriptomics has been used to study the effects of environmental variables on adult freshwater mussels [44–47], no study to date has used this technique with glochidia. In the present study, we provide the first comparison of gene expression in freshwater mussel glochidia metamorphosed in M199, L-15, and M199 supplemented with lipids.

## Materials and methods

### Extraction of glochidia

Three adult, female *Lampsilis siliquoidea* (Fatmucket) were collected from Big Darby Creek in Union County, Ohio on 28 September 2021. This species was chosen because it is common in Ohio, not listed by state or federal agencies, and has a relatively long brooding season [48]. Mussels were transported in coolers filled with water from the collection site to the Watters Aquatic Conservation Center (WACC) in Powell, Ohio. At the WACC, mussels were held in a streamside, flow-through, tank system supplied with water from the Scioto River and monitored for gravidity. On 28 April 2022, the gravid mussels were scrubbed clean with a toothbrush, gently opened with reverse pliers, and rinsed with sterile ultrapure water. Glochidia were extracted by piercing the marsupial gills with a 20 G hypodermic needle and flushing the glochidia out with sterile culture media to limit transfer of microorganisms [26]. A viability test was performed on a sub-sample of glochidia by adding a small amount of NaCl and calculating the percentage of glochidia that responded by closing their shells [49, 50]. Glochidia were cleaned of any debris, tissue residues, and eggs, by rinsing with sterile media through sieves of various sizes and then washed by swirling and decanting media from the beaker several times [26, 29, 51]. Open shells and individuals clamped onto one another were removed to prevent contamination. Glochidia were pooled prior to distribution into Petri dishes.

### Culture of glochidia

Pooled glochidia were separated into three treatment groups: M199 medium (M199), M199 medium supplemented with 50 μL of sterile lipid concentrate (M199 + lipids), and L-15 medium (L-15). The M199 group was chosen as the control because M199 is commonly and reliably used to metamorphose glochidia from a variety of species at the WACC and has been used in multiple other in vitro studies [22, 23, 52, 53]. Lipids are an important source of energy for young juveniles and the lipid content of diet has been correlated with juvenile growth [54]. Addition of lipids to culture media may increase lipid uptake by glochidia and improve their development and survival [20]. L-15 medium was chosen as an alternative to M199 since it has recently been used to successfully metamorphose freshwater mussel glochidia without CO_2_ supplementation [29], including species that were previously difficult to metamorphose using M199 [32].

Each treatment group consisted of 17 to 18 replicate dishes with approximately 1,000 glochidia per dish [29]. The culture media contained a volume ratio of 3:1 of basal medium (M199 or L-15) to rabbit serum (Sigma-Aldrich R4505). Media were prepared using powdered M199 (Sigma-Aldrich M0393) or L-15 (Sigma-Aldrich L4386) and sterile ultrapure water. Glutamic acid (Sigma-Aldrich PHR1107) was added to the L-15 medium per manufacturer’s instructions. Calcium hydroxide (0.1 M, Sigma-Aldrich 239,232) was added to both media to adjust pH to 7.65. Antibiotics and an antimycotic were added to prevent contamination and consisted of 5.33 mL of amphotericin B (0.25 mg/mL, Sigma-Aldrich A2942), 2.50 mL of carbenicillin (100 mg/mL, Sigma-Aldrich C1613), 13.33 mL of gentamicin (10 mg/mL, Sigma-Aldrich G1272), and 1.33 mL of rifampicin (100 mg/mL, Sigma-Aldrich R7382) per 1 L of media [23, 55, 56]. Media were sterilized by filtering through a 0.2 μm filter and stored in sterile conical tubes at −20 °C. Each dish (20 × 100 mm) contained 6 mL of M199 or L-15, 2 mL of rabbit serum, and 136 μL of antibiotics/antimycotic. Each dish in the M199 + lipids group was supplemented with 50 μL of lipid concentrate (Gibco 11905031).

All dishes were kept inside a CO_2_ incubator at 2.8% CO_2_ and 23 °C. Glochidia were placed into dishes once the media warmed to 23 °C to reduce stress from temperature fluctuations. If no contamination was observed, each dish received a 33% medium change every two to four days. If contamination was observed, a 100% medium change was performed by removing the glochidia from the dish, rinsing several times with new media, and placing the glochidia into a new dish with fresh medium. Glochidia developed in the incubator for 14 days, until most individuals exhibited signs of metamorphosis, which included foot movement outside of valves and observable gills with active cilia.

Metamorphosis rates were calculated by counting the number of metamorphosed individuals, dividing by the total initial number of glochidia added to the dish, and multiplying by 100. Typically, metamorphosis rates are calculated approximately 24–48 h after the culture medium is diluted with water, and our deviation from this typical procedure may impact our determination of metamorphosis success [51]. However, the decision to collect samples for transcriptome analysis at this point was based on the presumed high levels of DNA, RNA, and protein synthesis that have been found during this period [57]. Further examination of metamorphosis rates was conducted in a follow-up study (described below).

Significant differences in metamorphosis rates between the treatment groups were analyzed using one-way ANOVA with arcsine transformed metamorphosis percentages, and percent metamorphosis as a function of the type of culture media. Following the ANOVA test, Tukey’s honestly significant difference (HSD) test was performed to examine the pairwise differences among media types (α = 0.05), with metamorphosis success as the response variable. All statistical tests were conducted in RStudio (version 2021.09.0).

Juveniles from each dish were rinsed with sterile ultrapure water three times to remove any contaminants and residual media, placed in separate sterile cryogenic vials with RNAlater (Thermo Fisher Scientific AM7020), and snap-frozen in liquid nitrogen. Samples were stored at −80 °C and processed within two weeks.

### Transcriptome assembly and differential expression analysis

RNA was extracted using a Qiagen RNeasy Plus Micro RNA Extraction Kit (Qiagen 74034). RNA concentrations were measured using a Qubit Fluorometer 2.0 and a Thermo Scientific NanoDrop Spectrophotomer. RNA-Seq library preparation and sequencing were performed by The Ohio State University’s Comprehensive Cancer Center’s Genomic Shared Resource facility in Columbus, Ohio. RNA-Seq libraries were prepared using the NEBNext Ultra II Directional RNA Library Prep Kit for Illumina (NEB E7760L). Libraries were sequenced on the Illumina NovaSeq 6000 Sequences with output as 150 base pair, paired-end reads. Quality of sequencing data was assessed using FastQC (version 0.11.9; https://www.bioinformatics.babraham.ac.uk/projects/fastqc/).

Quality trimming was performed using Trimmomatic [58] in Trinity (version 2.15.1 [59],) with default parameters. Raw reads were scanned with a sliding window of four bases, trimmed when the average Phred quality score dropped below 5, which corresponds to a probability of an incorrect nucleotide call that is equal to 10^–5^, and discarded if shorter than 25 base pairs. De novo assembly of trimmed reads was performed using Trinity with default parameters. Redundant transcripts (with a default minimum similarity of 95%) were removed using CD-HIT (version 4.8.1; [60, 61]). The quality of the transcriptome was assessed using Bowtie2 (version 2.4.1), which calculates the percentage of raw reads used in the transcriptome assembly [62]. Transcriptome completeness was assessed using the metazoan lineage in BUSCO (version 5.4.7), which calculates the percentage of completely assembled, highly conserved, single-copy orthologs [63].

Transcripts were used as BLASTx (version 2.13.0) queries against the National Center for Biotechnology Information nonredundant database, with an E-value cutoff of 1e^−5^ (the number of matches expected to occur by chance) and a hit threshold of 20. Functional annotation of transcripts using Gene Ontology (GO) terms was performed using OmicsBox (version 2.2.4; [64]) with default parameters.

Transcript expression was quantified using Salmon (version 1.10.0; [65]). Differential expression analysis was performed using EdgeR (version 4.0.16; [66]) with a False Discovery Rate (FDR) of 0.001 and a minimum fold-change of four. Significantly overrepresented GO terms of the differentially expressed transcripts between groups were identified using a two-tailed Fisher’s Exact Test [67] in combination with a Benjamin-Hochberg FDR correction (p-adj = 0.05) using OmicsBox. Results from the Fisher’s Exact Test were reduced and summarized using REVIGO (http://revigo.irb.hr, [68]).

### Follow-up study

A follow-up study was conducted to look specifically at the post-dilution metamorphosis rates of *L. siliquoidea* reared in the various culture media types. One adult, female *L. siliquoidea* was collected from the Cuyahoga River in Portage County, Ohio on 16 May 2022 and transported to the WACC. On 6 June 2024, glochidia were extracted from the mussel using the methods described above. Glochidia were separated into 12 replicate dishes containing M199, 6 dishes containing M199 supplemented with lipid concentrate, and 6 dishes containing L-15. The average number of glochidia per dish was less than 100. Culture media preparations and subsequent incubation methods were the same as for the previous study. Metamorphosed juveniles were counted post-dilution on day 16.

## Results

### Metamorphosis rates

Metamorphosis rates for M199, L-15, and M199 + lipids were 99 ± 1%, 98 ± 1%, and 99 ± 1%, respectively. Although all metamorphosis rates were similar and very high, one-way ANOVA indicated that culture media type did have a significant effect on metamorphosis rates (F_2,56_ = 4.77; p = 0.01). Tukey’s HSD test revealed that metamorphosis rates were significantly lower in L-15 than in M199 (p = 0.01), but not significantly different between M199 and M199 + lipids (p = 0.61) or between M199 + lipids and L-15 (p = 0.10).

In the follow-up study, metamorphosis rates for M199, L-15, and M199 + lipids were 71 ± 2%, 53 ± 1%, and 70 ± 3%, respectively. When M199 + lipids and L-15 were compared to M199 separately using a two-tailed t-test, there were no significant differences in metamorphosis rates between M199 and M199 + lipids (p = 0.90), but metamorphosis in L-15 was significantly lower (p = 0.02) than in M199. However, when the three treatment groups were analyzed collectively using ANOVA, no significant differences between groups were found (F_2,21_ = 1.33; p = 0.29).

### Transcriptome assembly

Illumina sequencing produced 2,714,275,948 reads. Trimmed, high quality reads were assembled into 1,830,351 transcripts with an average length of 504 bp, a median transcript length (N50) of 571 bp, and a guanine-cytosine (GC) content of 43.28% (Table 1). Bowtie2 calculated a 97.87% read alignment to the transcriptome assembly. BUSCO analysis indicated that the assembly produced 948 (99.3%) complete, 6 (0.6%) fragmented, and 0 missing BUSCOs. Data were archived in GenBank under BioProject accession number PRJNA1261908.

**Table 1:** Summary statistics for sequencing and transcriptome assembly. N50 = 50% of transcripts are equal to or larger than this value; bp = base pairs; GC = guanine-cytosine.

Statistic	Value
Raw reads produced by Illumina sequencing	2,714,275,948
Estimate of reads used in final assembly	97.87%
Total assembled transcripts	1,830,351
Total assembled bases	814,024,969
Mean transcript length	504 bp
Median transcript length	309 bp
N50	571 bp
GC content	43.28%

**Fig. 1:**
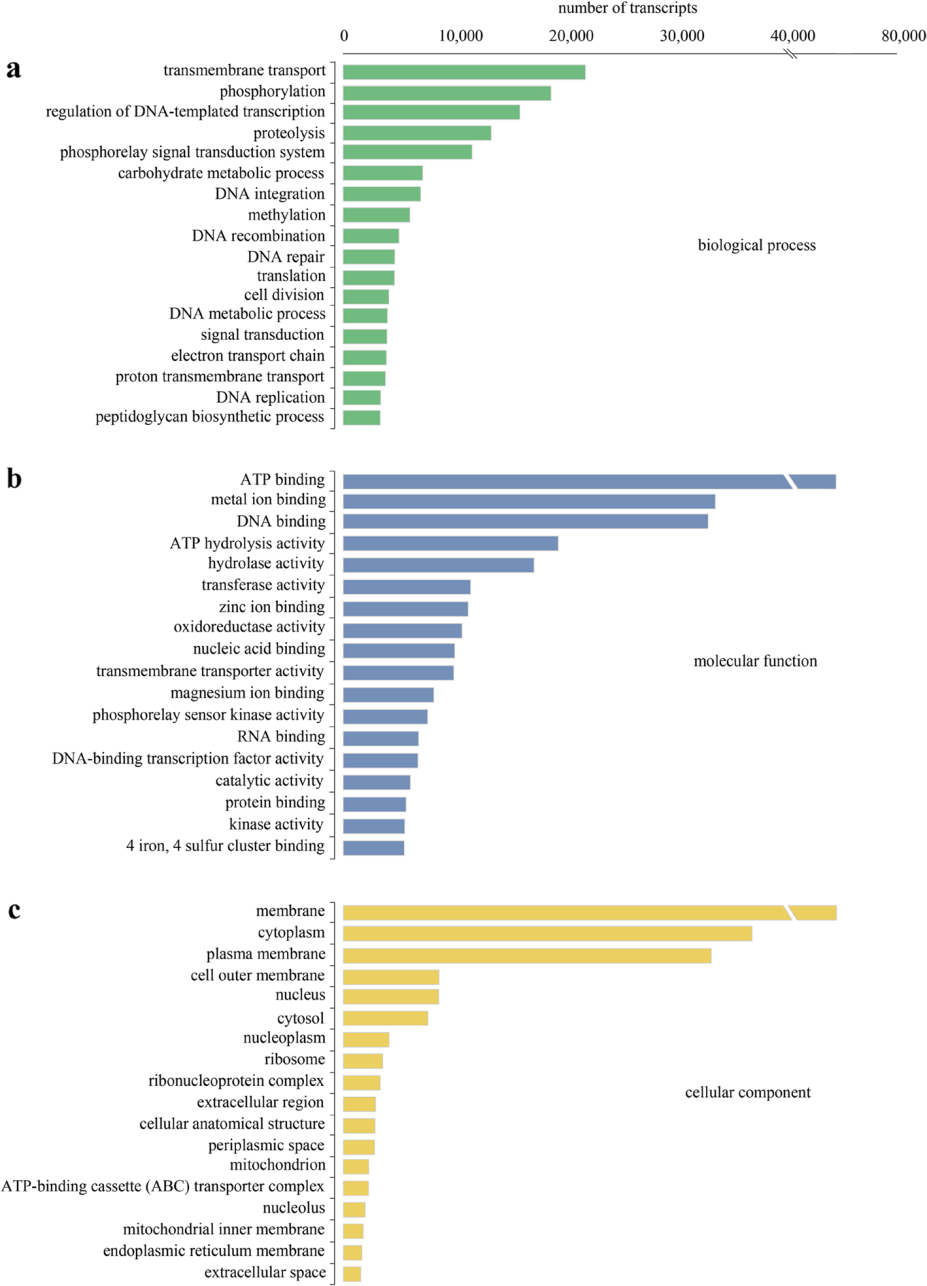
Number of transcripts assigned to Gene Ontology terms from the “biological process” (**a**), “molecular function” (**b**), and “cellular component” (**c**) domains

Of the 1,830,351 transcripts assembled by Trinity, 627,072 (34.26%) received BLAST hits, and 399,248 (21.81%) were annotated with GO terms. Transcripts were assigned to one or more of the three primary GO domains: “biological process” “molecular function” and “cellular component”. The categories within the biological process domain that had the highest number of transcripts were “transmembrane transport”, “phosphorylation”, “regulation of DNA-templated transcription”, and “proteolysis” (Fig. 1a). The most common categories within the molecular function domain were “ATP binding”, “metal ion binding”, “DNA binding”, and “ATP hydrolysis activity” (Fig. 1b), and those in the cellular component domain were “membrane”, “cytoplasm”, “plasma membrane”, and “cell outer membrane” (Fig. 1c).

### Differential expression

Compared to the standard M199 culture medium, differential expression was detected in 22,532 transcripts in the L-15 treatments, and in 22,867 transcripts in the M199 + lipids treatments. The vast majority of differentially expressed transcripts were downregulated in both treatment groups: 22,150 in L-15, and 22,536 in M199 + lipids. Only a small portion were upregulated: 382 in L-15, and 331 in M199 + lipids. Among the downregulated transcripts, 19,545 (87–88%) transcripts were identical between the two treatment groups. However, among the upregulated transcripts, only 40 (10–12%) were identical.

**Fig. 2:**
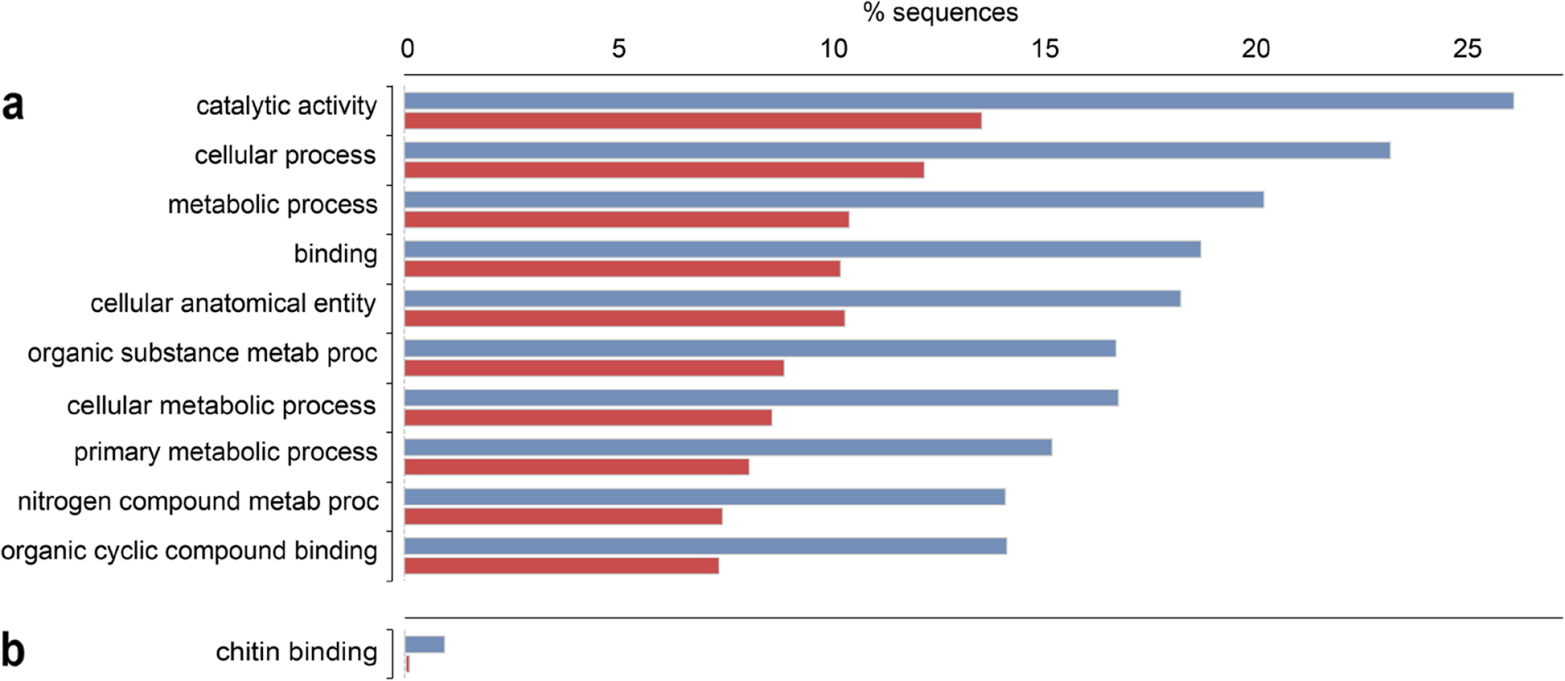
Overrepresented GO terms for (**a**) downregulated and (**b**) upregulated transcripts from glochidia transformed in L-15 medium (L15) compared to M199 medium. Top (blue) bars represent the entire transcriptome. Bottom (red) bars represent the differentially expressed subset

**Fig. 3:**
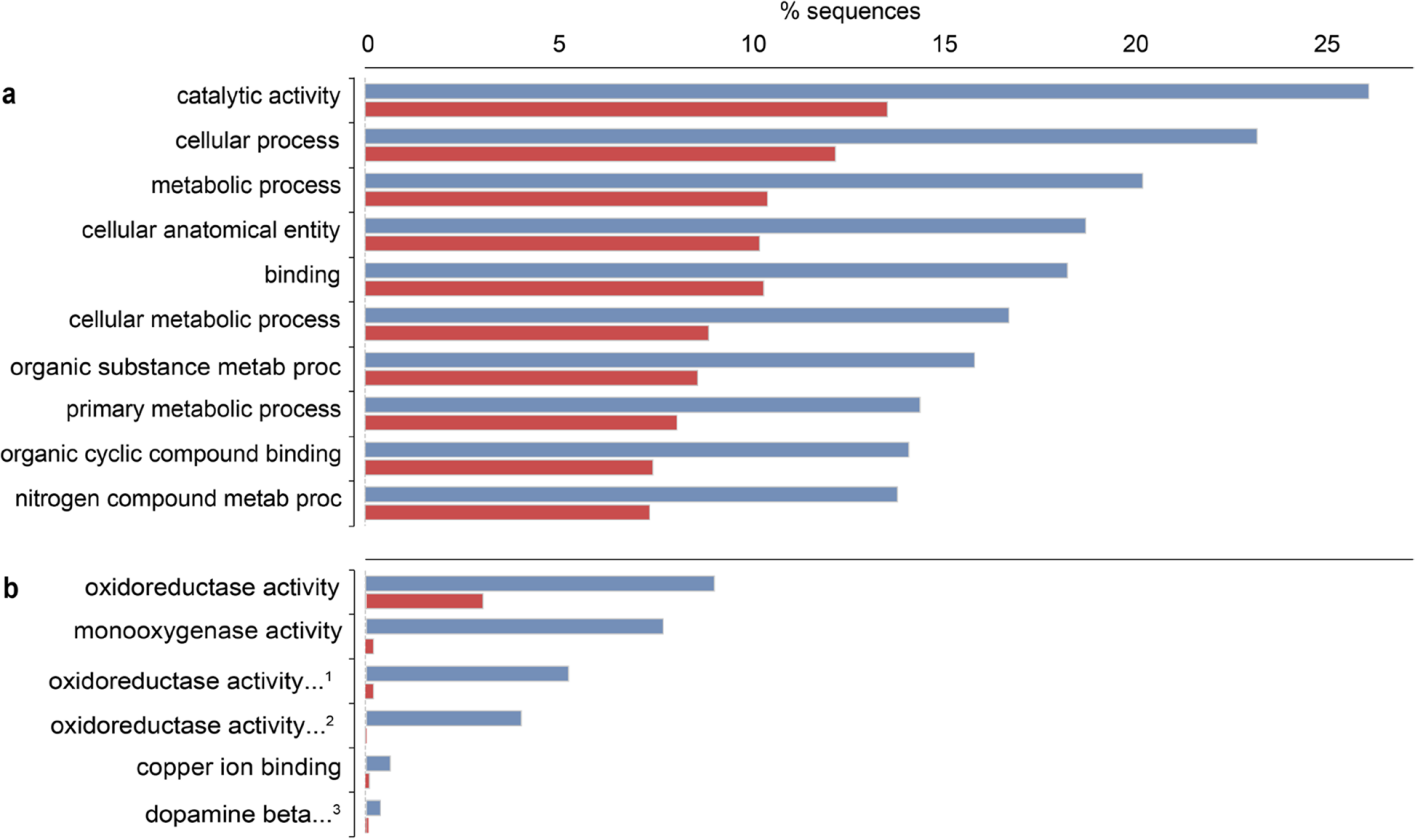
Overrepresented GO terms for (**a**) downregulated and (**b**) upregulated transcripts from glochidia transformed in M199 medium supplemented with lipids (M199 + lipids) compared to M199 medium. Top (blue) bars represent the entire transcriptome. Bottom (red) bars represent the differentially expressed subset. ^1^Oxidoreductase activity, acting on paired donors, with incorporation or reduction of molecular oxygen. ^2^Oxidoreductase activity, acting on paired donors, with incorporation or reduction of molecular oxygen, reduced ascorbate as one donor, and incorporation of one atom of oxygen. ^3^Dopamine beta-monooxygenase activity

Due to the high number of differentially expressed transcripts that were shared between the two treatment groups, the overrepresented GO terms among differentially expressed transcripts were also similar (Tables 2 and 3). Top overrepresented GO terms with the most associated transcripts were similar for both L-15 and M199 + lipids: “catalytic activity”, “cellular process”, “metabolic process”, “binding”, and “cellular anatomical entity” (Fig. 2 and 3).

**Table 2:** GO accession numbers and terms overrepresented in the L-15 treatment compared to the standard M199 culture medium. Overrepresented GO terms were identified using a Fisher’s Exact Test and reduced and summarized using REVIGO. Bolded GO terms are identical to those overrepresented in the M199 + lipids treatment (see Table 3)

BIOLOGICAL PROCESS	MOLECULAR FUNCTION	CELLULAR COMPONENT
*L-15 downregulated*		
**0065007 Biological regulation**	0022804 Active transmembrane transporter activity	**0030313 Cell envelope**
**0015977 Carbon fixation**	**0033218 Amide binding**	**0071944 Cell periphery**
**0007154 Cell communication**	**0016209 Antioxidant activity**	**0110165 Cellular anatomical entity**
**0051301 Cell division**	**0140657 ATP-dependent activity**	**0005622 Intracellular anatomical structure**
**0071554 Cell wall organization or biogenesis**	**0005488 Binding**	**0016020 Membrane**
**0071840 Cellular component organization or biogenesis**	0016830 Carbon–carbon lyase activity	**0019867 Outer membrane**
**0009987 Cellular process**	**0003824 Catalytic activity**	**0042597 Periplasmic space**
0051276 Chromosome organization	0140097 Catalytic activity, acting on DNA	**0032991 Protein-containing complex**
**0006091 Generation of precursor metabolites and energy**	**0009975 Cyclase activity**	**0098803 Respiratory chain complex**
**0042592 Homeostatic process**	**0009055 Electron transfer activity**	
0006879 Intracellular iron ion homeostasis	0051536 Iron-sulfur cluster binding	
**0051179 Localization**	0051003 Ligase activity, forming nitrogen-metal bonds	
**0040011 Locomotion**	**0140299 Molecular sensor activity**	
**0008152 Metabolic process**	**0060089 Molecular transducer activity**	
**0006811 Monoatomic ion transport**	**0016675 Oxidoreductase activity, acting on a heme group of donors**	
**0006518 Peptide metabolic process**	**0004673 Protein histidine kinase activity**	
**0000160 Phosphorelay signal transduction system**	0016987 Sigma factor binding	
**0046148 Pigment biosynthetic process**	**0038023 Signaling receptor activity**	
**0050896 Response to stimulus**	**1,901,681 Sulfur compound binding**	
**0032196 Transposition**	**0140110 Transcription regulator activity**	
	**0005215 Transporter activity**	
*L-15 upregulated*		
	GO:0008061 Chitin binding

**Table 3:** GO accession numbers and terms overrepresented in the M199 + lipids treatment compared to the standard M199 culture medium. Overrepresented GO terms were identified using a Fisher’s Exact Test and reduced and summarized using REVIGO. Bolded GO terms are identical to those overrepresented in the M199 + lipids treatment (see Table 2)

BIOLOGICAL PROCESS	MOLECULAR FUNCTION	CELLULAR COMPONENT
*M199* + *lipids downregulated*		
**0065007 Biological regulation**	**0033218 Amide binding**	0044297 Cell body
**0015977 Carbon fixation**	0001540 Amyloid-beta binding	**0030313 Cell envelope**
**0007154 Cell communication**	**0016209 Antioxidant activity**	**0071944 Cell periphery**
**0051301 Cell division**	**0140657 ATP-dependent activity**	0042995 Cell projection
0042546 Cell wall biogenesis	0016887 ATP hydrolysis activity	**0110165 Cellular anatomical entity**
**0071554 Cell wall organization or biogenesis**	**0005488 Binding**	0030312 External encapsulating structure
**0071840 Cellular component organization or biogenesis**	**0003824 Catalytic activity**	0005576 Extracellular region
**0009987 Cellular process**	0005507 Copper ion binding	0005616 Extracellular space
0048878 Chemical homeostasis	**0009975 Cyclase activity**	**0005622 Intracellular anatomical structure**
0007059 Chromosome segragation	0097718 Disordered domain specific binding	**0016020 Membrane**
**0006091 Generation of precursor metabolites and energy**	0003700 DNA-binding transcription factor activity	0005739 Mitochondrion
**0042592 Homeostatic process**	**0009055 Electron transfer activity**	0043209 Myelin sheath
0007611 Learning or memory	0005539 Glycosaminoglycan binding	0043025 Neuronal cell body
**0051179 Localization**	0020037 Heme binding	0043226 Organelle
**0040011 Locomotion**	0016866 Intramolecular transferase activity	**0019867 Outer membrane**
**0008152 Metabolic process**	0016875 Ligase activity, forming carbon–oxygen bonds	**0042597 Periplasmic space**
**0098655 Monoatomic cation transmembrane transport**	0072341 Modified amino acid binding	**0032991 Protein-containing complex**
**0006518 Peptide metabolic process**	0140104 Molecular carrier activity	**0098803 Respiratory chain complex**
**0000160 Phosphorelay signal transduction system**	**0140299 Molecular sensor activity**	0036477 Somatodendritic compartment
**0046148 Pigment biosynthetic process**	**0060089 Molecular transducer activity**	0045202 Synapse
**0050896 Response to stimulus**	**0016675 Oxidoreductase activity, acting on a heme group of donors**	
**0032196 Transposition**	0015453 Oxidoreduction-driven active transmembrane transporter activity	
	0042277 Peptide binding	
	**0004673 Protein histidine kinase activity**	
	**0038023 Signaling receptor activity**	
	0019911 Structural constituent of myelin sheath	
	**1,901,681 Sulfur compound binding**	
	**0140110 Transcription regulator activity**	
	**0005215 Transporter activity**	
*M199* + *lipids upregulated*		
	0005507 Copper ion binding	
	0016491 Oxidoreductase activity

### L-15 vs. M199

A total of 22,150 transcripts were downregulated in glochidia metamorphosed in L-15, compared to glochidia in M199. Of those transcripts, 11,811 received BLAST hits and 8,106 were annotated. Among the downregulated transcripts, 748 GO terms were overrepresented, with most associated with “catalytic activity”, “cellular process”, and “metabolic process” (Fig. 2a), same as for M199 + lipids (Fig. 3a). The overrepresented GO terms that were unique to the L-15 treatment included those involved in iron homeostasis, transmembrane transporter activity, carbon–carbon lyase activity, and sigma factor binding (Table 2).

A total of 382 transcripts were upregulated in glochidia metamorphosed in L-15, compared to glochidia in M199. Of those transcripts, 190 received BLAST hits and 43 were annotated. Among the upregulated transcripts, only one GO term, “chitin binding”, was overrepresented (Fig. 2b), and was also unique to the L-15 treatment (Table 2).

### M199 + lipids vs. M199

A total of 22,536 transcripts were downregulated in glochidia metamorphosed in M199 + lipids, compared to glochidia in M199. Of those transcripts, 10,933 received BLAST hits and 8,214 were annotated. Among the downregulated transcripts, 923 GO terms were overrepresented, with most associated with “catalytic activity”, “cellular process”, and “metabolic process” (Fig. 3a), same as for L-15 (Fig. 2a). The overrepresented GO terms that were unique to the M199 + lipids treatment included several related to the structure and function of neurons, such as “learning or memory”, “amyloid-beta binding”, “myelin sheath”, “somatodendritic compartment” and “synapse” (Table 3).

A total of 331 transcripts were upregulated in glochidia metamorphosed in M199 + lipids, compared to glochidia in M199. Of those transcripts, 232 received BLAST hits and 99 were annotated. Among the upregulated transcripts, six GO terms were overrepresented, mainly related to oxidoreductase activity (Fig. 3b), and were also unique to the M199 + lipids treatment (Table 2).

## Discussion

To the best of our knowledge, this was the first study to look at gene expression in freshwater mussel glochidia. We provide a transcriptome resource of genes that are involved in metamorphosis (Online Resource) and highlight the biological processes that are particularly impacted by changes in the external environment, i.e., culture medium (Tables 2 and 3). Likely due to the metabolic demands of metamorphosis, our transcriptome assembly produced more transcripts than typically found in studies of adult freshwater mussels [44, 46, 47, 69–71]. Approximately 1.2% of the transcripts expressed during metamorphosis had significantly altered expression levels in response to different types of culture media. Of these differentially expressed transcripts, 86% were the same regardless of whether glochidia were metamorphosed in a different basal culture medium (L-15 instead of M199) or in the same basal culture medium (M199) supplemented with lipids.

Due to the high percentage of downregulated transcripts that were shared between the L-15 and M199 + lipids treatments, the lists of overrepresented GO terms were also similar (Tables 2 and 3). As expected during metamorphosis, the biological processes impacted by changes in culture medium were related to cell replication and growth, such as “cell division”, “generation of precursor metabolites and energy”, “catalytic activity”, “transcription regulator activity”, and many others. These biological processes likely reflect key pathways in glochidia metamorphosis that are particularly sensitive to environmental changes. The few GO terms that were unique to each treatment may represent pathways that are more responsive to select stressors.

Pathways involved in energy metabolism were mostly down-regulated in both the L-15 and M199 + lipids treatments. Examples included “cell division”, “generation of precursor metabolites and energy”, and “metabolic process” (Tables 2 and 3). Metamorphosis for most organisms is typically considered to be a costly and energy-demanding process [72–74], so the down-regulation of metabolism suggests a less favorable culture environment, which is particularly surprising for the M199 + lipids treatment. However, we did notice lipid layers floating on top of the culture medium in some dishes, so it is possible that addition of lipids could reduce gas permeability, thereby affecting metabolic pathways. Future studies using various lipid concentrations could provide more insight on how to balance potential positive and negative effects of lipid additions.

Glochidia cultured in both L-15 and M199 + lipids also had decreased expression of transcripts related to the stress response, including the universal stress protein and the heat shock proteins HSP90 and HSP33 (Online Resource). Because HSPs are frequently up-regulated in response to various environmental stressors [75–77], it is possible that the down-regulation of these genes in the L-15 and M199 + lipids treatments suggests a less stressful environment. However, some studies have indicated that HSPs play important roles during larval metamorphosis of ascidians [78] and the common cutworm [79] and during development of the vegetable leafminer, *Liriomyza sativae* [80]. Since HSPs and other molecular chaperones assist in the conformational folding and assembly of other proteins during protein synthesis and translocation [81], it may be that HSPs play roles in ensuring successful cell replication and growth during metamorphosis of freshwater mussel glochidia, in which case, their down-regulation might not be ideal.

### L-15 vs. M199

Both M199 and L-15 culture media are designed to support cell growth in a laboratory setting. However, the formulations of the two media differ in the exact types and concentrations of organic salts, amino acids, vitamins, and other compounds. The amino acid composition between the two media is similar, though L-15 generally has higher concentrations. Similarly, for vitamins, concentrations tend to be higher in L-15, but variety is greater in M199. In total, M199 is enriched with 61 compounds, while L-15 contains only 35 compounds.

Although the pre-dilution metamorphosis rates calculated in this study were all very high (98–99%), the rates for L-15 were statistically significantly lower (98%) than those for M199 (99%), albeit minimally. However, the post-dilution rates, which are considered more accurate representations of metamorphosis success [51], calculated in our follow-up study, indicated that metamorphosis success is lower in L-15 (53%) than in M199 (71%) when analyzed separately using t-tests, but not when analyzed collectively using ANOVA. Together, these results suggest that M199 may be the most suitable culture medium for transforming *L. siliquoidea*. Although Wen et al. [32] discovered that L-15 could be used to metamorphose *P. alatus*, which up to that point had not been accomplished using M199, our study shows that M199 may still be the better culture medium choice for other species. However, further studies using more replicates would help form a stronger conclusion.

“Sigma factor binding” was an overrepresented GO term among downregulated transcripts that was unique to the L-15 treatment (Table 2). Sigma factors are bacterial proteins that bind to RNA polymerase and are needed for initiation of transcription [82]. Because sigma factors do not occur in eukaryotes, their presence in the transcriptome assembly indicates bacterial contamination, which is inevitable to some extent during in vitro mussel propagation. However, because these transcripts were more downregulated in the L-15 treatment, this could suggest that the L-15 dishes had a lower bacterial load or that the L-15 medium may be less conducive to bacterial replication.

“Chitin binding” was the only overrepresented GO term among upregulated transcripts and was also unique to the L-15 treatment (Table 2). Bivalve shells are composed of inorganic CaCO_3_ and an organic matrix of proteins and polysaccharides, including chitin [83, 84]. The upregulation of transcripts involved in chitin formation was surprising because the shell size of *L. siliquoidea* does not change during metamorphosis [85] and chitin itself likely makes up only a small portion of bivalve shell material [86].

### M199 + lipids vs. M199

Lipid supplementation of M199 culture medium did not alter metamorphosis rates, but the changes that occurred in the transcriptome were equivalent in scope as for the L-15 treatment. Contrary to our expectations, we did not see an overrepresentation of GO terms related directly to lipid metabolism (Table 3). Instead, many GO terms unique to the M199 + lipids treatment were related to the structure and function of neurons. Although we expected lipid supplementation to impact energy metabolism, it is not surprising that transcription of genes involved in neuronal development was altered since lipids are important components of the cell membrane, function as signaling molecules, and can regulate neural cell development [87–89].

Lipids are an important energy source for organisms, and most of the recent in vitro studies of freshwater mussels have included the addition of a lipid source. However, lipid supplementation has not always yielded similar results. The elimination of a lipid source significantly reduced the glochidia metamorphosis rates of the New Zealand freshwater mussel *Echyridella menziesii* [90], indicating the importance of a lipid source for successful larval metamorphosis. Switching from traditional fish oil to an emulsified lipid mixture significantly increased survival rates of *Margaritifera margaritifera* glochidia [34]. However, Owen [91] found no statistically significant differences in metamorphosis rates of *Utterbackia imbecillis* glochidia propagated with and without lipid supplementation. The addition of lipids to M199 in our study also did not result in significant differences in metamorphosis rates, but did cause differential gene expression, suggesting that lipids play an important physiological role in developing glochidia and may impact successful propagation.

Down-regulation of transcripts involved in energy metabolism in the L-15 treatment was particularly surprising, since lipids are often the primary energy source used during metamorphosis in other invertebrates, such as the barnacle *Balanus amphitrite* [92], the seastar *Mediaster aequalis* [93], and the fruit fly *Drosophila melanogaster* [94]. However, Rodriguez et al. [95] found that the larvae of the European flat oyster, *Ostrea edulis*, mainly utilized protein during metamorphosis. A further investigation into the energy sources utilized by freshwater mussel glochidia is warranted and may be better understood by observing survival and growth rates of juveniles post metamorphosis.

## Conclusion

We have provided the first transcriptomic resource for metamorphosing freshwater mussel glochidia and highlight the physiological pathways that are altered in response to culturing in different types of media. However, there remains much room for discovery, as more than half of the transcripts were not identified through BLAST. Our study also further supports the unique responses of individual freshwater mussel species to propagation techniques. Although L-15 may be the choice of culture media for some species such as *P. alatus* [32], the more typically used M199 may still be the better choice for other species such as *L. siliquoidea*. The significant differential gene expression observed in glochidia cultured in different media also suggests that the physiology of metamorphosing freshwater mussels may be impacted by the host fish species that they use. Even among freshwater mussel species that can use a variety of host fish species, differences have been observed in metamorphosis rates depending on whether the fish species used is considered a primary or secondary host [96]. Our study suggests that the type of serum used might also impact the physiology of the transforming glochidia and, therefore, potentially the health and survival of juveniles.

## Supplementary Information

Supplementary Material 1.

## Data Availability

Data are archived in GenBank under BioProject accession number PRJNA1261908.
